# The Effect of Species and Sex on the Element Content of Muskox (*Ovibos moschatus*) and Caribou (*Rangifer tarandus groenlandicus*) Tissues

**DOI:** 10.1007/s12011-023-03562-x

**Published:** 2023-01-17

**Authors:** David Miguel Ribeiro, Katrine Raundrup, Miguel P. Mourato, André M. Almeida

**Affiliations:** 1 LEAF—Linking Landscape, Environment, Agriculture and Food Research Center, Associated Laboratory TERRA, Instituto Superior de Agronomia, Universidade de Lisboa, Tapada da Ajuda, 1349-017 Lisbon, Portugal; 2 Greenland Institute of Natural Resources, Kivioq 2, P.O. Box 570, 3900 Nuuk, Greenland

**Keywords:** Muskox, Caribou, Sex, Tissue elements

## Abstract

Muskox (*Ovibos moschatus*) and caribou (*Rangifer tarandus groenlandicus*) are wild ruminants that inhabit the Greenland tundra. They are part of the diet of many Greenlanders, being important sources of protein and micronutrients such as iron. The objective of this study is to analyse the element profiles of three tissues from these species: skeletal muscle, liver and adipose tissue, and to determine if they are affected by species and sex (male vs. female). Samples were obtained from annual hunts in two different regions of West Greenland. Element profiles were analysed using inductively-coupled plasma–optical emission spectrometry. The interaction between species and sex was only detected in Na (sodium) in the muscle and adipose tissue, where male and female caribou had the highest concentrations, respectively. The effect of sex was not statistically significant in the liver samples and only occasionally in the other tissues. Species was the most relevant factor in element profiles found in this study. Caribou had higher concentrations of K (potassium) and S (sulphur) in the muscle and liver. Fewer differences were detected between species in the adipose tissue, compared to the other tissues. These differences may reflect the feeding behaviour and the geographical location of both species. This study contributes to evaluate the element composition of the edible tissue of these wild ungulate species, as well as evaluating the factors of sex and species that could differentiate their composition.

**Supplementary Information:**

The online version contains supplementary material available at 10.1007/s12011-023-03562-x.

## Introduction

**Fig. 1:**
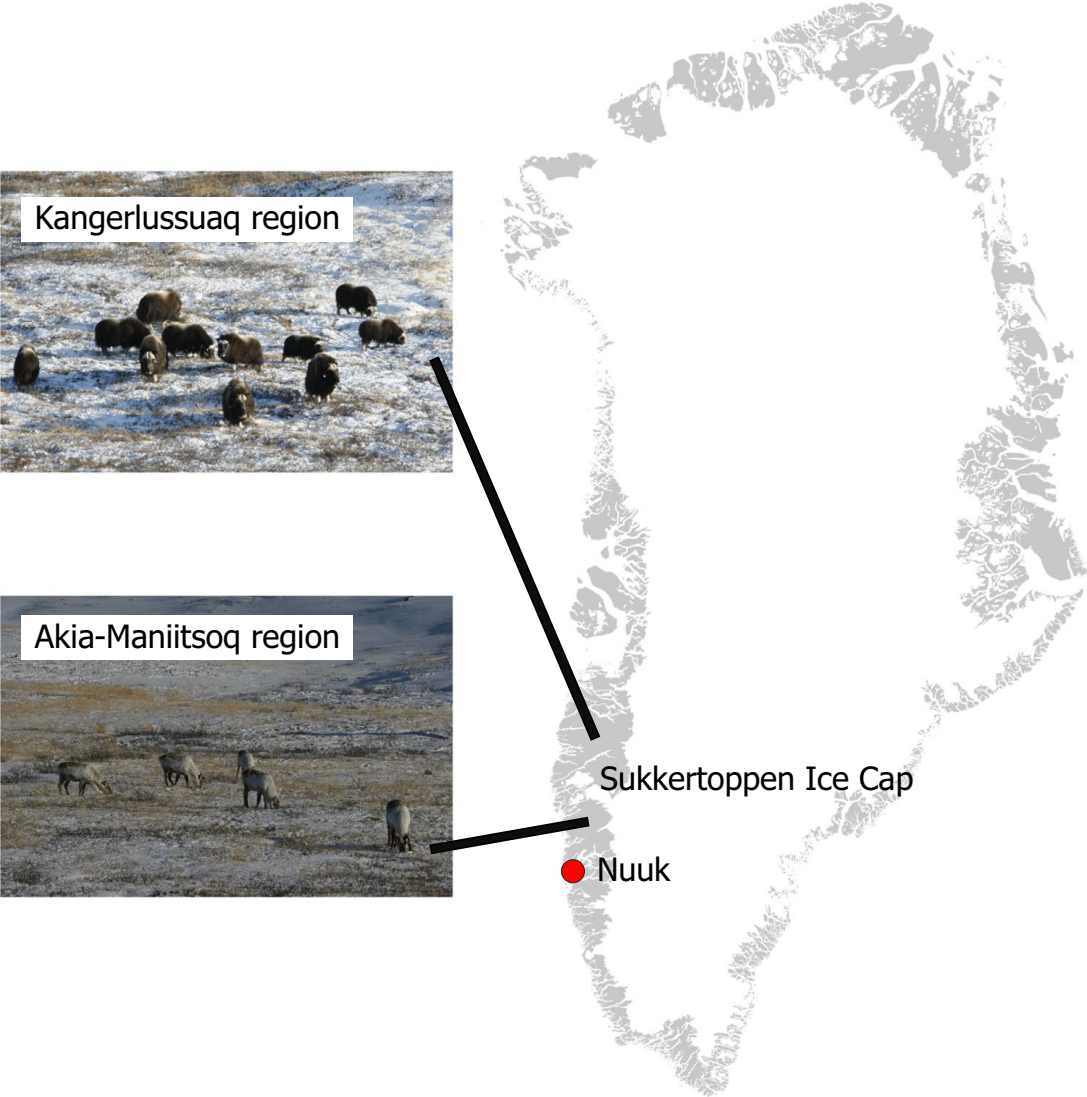
Geographical location of the muskox and caribou populations (made using QGIS)

The Arctic has long been the habitat of two wild ungulate species: the muskox (*Ovibos moschatus*) and the caribou (*Rangifer tarandus groenlandicus*). These species co-exist in West Greenland in the Kangerlussuaq-Sisimiut (KS) region, while only caribou are found in the Akia-Maniitsoq (AM) region (Fig. 1). During the latest survey in 2018, the muskox population in the KS region was estimated at ca. 20.300 animals [1]. while caribou numbers in the KS and AM regions were estimated at ca. 98.300 animals and 24.000, respectively [2]. Both species need to accumulate nutrients during the spring and summer, when forage quality and quantity are the highest. The muskox is predominantly a grazer, whereas the caribou is an intermediate feeder [3]. In addition, the caribou feeds on lichens during the winter if they are available [3]. The muskox has developed a slower digestive process, maximizing feed intake, enabled by a large rumen compared to that of the caribou [4]. The element status of wild animals is influenced by forage availability and their feeding behaviour, ultimately influencing their health and meat quality [5].

Elements are of paramount importance for the maintenance of physiological functions. Herein, these will be divided in three classes: macroelements (e.g. sodium (Na), potassium (K), sulphur (S)), microelements (e.g. iron (Fe), zinc (Zn), copper (Cu)) and other non-essential elements (e.g. cadmium (Cd), arsenic (As) and lead (Pb)). Their functions range from maintaining acid–base balance to acting as key structural components of molecules and organisms [5]. Several factors influence element concentration in tissues, including nutrition, geographic location and sex. Feed restriction has been reported to increase element concentrations in the muscle, liver and adipose tissue of ram lambs (*Ovis aries*) [6, 7]. Sex influences the element concentration of tissues from large ruminant species, such as the yak (*Bos grunniens*) or water buffalo (*Bubalus bubalis*) [5]. The influence of nutrition and geographic location has been described in the liver and kidney tissues of Svalbard reindeer (*Rangifer tarandus platyrhynchus*), reflecting the impact of different dietary compositions in distinct populations [8]. In Canada, the concentrations of selenium (Se) in the liver and serum of muskox were found to be low, which was attributed to the geographic location and soil properties [9]. The objective of this study is to analyse the effect of sex and species on the element content of muscle, liver and fat of caribou and muskox from West Greenland. This will provide complementary information to existing literature on the effect of sex as well as information on elements (including contaminants) that may be present in such edible tissues, focusing on the two specific regions of Greenland.

## Materials and Methods

- a. Sample Collection

Procedures for sample harvesting have been described for the muskox samples [10, 11]. Briefly, muskoxen were hunted in the Kangerlussuaq-Sisimiut region, West Greenland during winter. Animals were eviscerated and bled on kill site and brought to the Kangerlussuaq butchery where samples were taken from the *longissimus dorsi* muscle, liver and subcutaneous adipose tissue of female (*n* = 12) and male (*n* = 8) muskoxen. Average carcass weight of the sampled animals was 76.0 ± 9.51 and 89.5 ± 19.87 kg for females and males, respectively. The caribou samples were harvested in a similar manner, during an annual summer hunt in the Akia-Maniitsoq region. Samples were taken from ten males and eight females on site. All samples were frozen, transported to the lab and freeze-dried for 72 h until constant weight using a Christ Alpha 1-2 LD_plus_ freeze drier (Christ Alpha, Osterode am Harz, Germany). They were then shipped to Portugal for further analysis.

Samples from both species were obtained during the annual commercial hunting season following Greenland legislation. Animals were thus hunted for consumption and not for research purposes. As such, no ethical approval is necessary.

- b. Element Analysis

The protocol for element analysis was previously described in Ribeiro et al. [6]. Briefly, approximately 0.3 g of dried sample was weighed into a digestion tube. For sample dissolution, hydrogen peroxide, nitric acid and hydrochloric acid — 1:3:10 (v/v/v) — were added. Each digestion set had a blank tube, without sample, to perform blank correction. The tubes were randomly distributed in a digestion plate (DigiPREP MS, SCP Science, Quebec, Canada) in which they were heated gradually for 1 h until reaching 95 °C and then at 95 °C constantly for another hour. Samples were then diluted to a total volume of 25 mL using distilled water and filtered using 90-mm filter paper (Filter-Lab ref. 1242, Filtros Anoia S.A., Barcelona, Spain).

Inductively coupled plasma–optical emission spectrometry (ICP-OES) readings were performed in a Thermo Scientific iCAP 7200 Duo spectrometer (Thermo Scientific, Waltham, MA, USA). Multi-element PlasmaQual S22 standards (SCP Science, Baie D’Urfé, QC, Canada) were used to create the calibration curves necessary to quantify the different elements (between 5 and 300 mg/L for Na, K, Ca, Mg, P and S and between 0.05 and 20 mg/L for the other elements). Multi-element detection and quantification took place overnight to detect the following 21 elements (limits of quantification, in mg/kg, are indicated after the name of the element): Sn (tin, 2.5), V (vanadium, 0.5), Li (lithium, 1.0), Ba (barium, 2.0), Se (selenium, 1.0), As (arsenic, 2.0), Co (cobalt, 1.0), Zn (zinc, 1.0), Fe (iron, 1.0), Mn (manganese, 1.0), Cu (copper, 1.0), Pb (lead, 0.5), Cd (cadmium, 0.05), Ni (nickel, 2.0), Cr (chromium, 2.0), S (sulphur, 50), P (phosphorous, 50), Mg (magnesium, 20), Ca (calcium, 20), K (potassium, 150) and Na (sodium, 150). Recovery values from spiked samples were always within a 10% range. Results were confirmed by quantifying the elements in certified reference materials (Wepal IPE 776) that underwent the same experimental procedure as the samples. Recovery values of the standards are reported in supplementary material 1. No further dilution was needed for any element before analysis. Results were calculated on a dry matter (DM) basis without correcting for lipid content.

- c. Statistical Analysis

In total, 18 elements were identified in all tissues: Na, K, Ca, Mg, P, S, Cr, Cd, Pb, Cu, Mn, Fe, Zn, Co, As, Ba, Li and Sn. Three elements were identified in concentrations below the lower limit of quantification and were not considered for subsequent statistical analysis: Ni (< 2.0 mg/kg DM), Se (< 1.0 mg/kg DM) and V (< 0.5 mg/kg DM). Whenever a reading was found to be below the limit of quantification, it was considered as not acquired and was not used to calculate means. Analysis of variance was carried out using the GLM procedure of SAS system, 3rd edition (SAS Institute Inc.): species, sex and the interaction between the two factors were fitted. When significant *p* values (*p* < 0.05) of the interaction were obtained, least square means were compared using the Tukey test. The univariate procedure was used to obtain standard error of the means (SEM). A principal component analysis (PCA) was carried out using the *prcomp* function and the *factoextra* package in R Studio (version 3.6.2, The R Foundation for Statistical Computing) to visualize the first two components.

## Results and Discussion

In this study, like others where samples are collected from field conditions, differences found with statistical significance may have a multitude of causes. There are differences inherent to each species: the caribou has more selective feeding habits, while the muskox feeds in bulk and has a larger rumen, comparatively increasing digestive efficiency of poor-quality forage [4].

Element concentrations of the muscle tissue are presented in Table 1, and the corresponding *p*-values for their effects are depicted in Table 2. At the species level, caribou had higher concentrations of K, Mg, P and S than muskox, reaching a 1.95-fold difference in S (*p* < 0.05). The contrary was observed for Ca, where muskox had higher concentrations. In the liver (Tables 3 and 4), all macroelements had statistically significant differences between species (*p* < 0.05). Muskox had higher concentrations of Na, Ca and Mg, whereas caribou had higher concentrations of K, P and S. Caribou had higher concentration of Mn compared to muskox, whereas the concentration of Cu had the inverse relationship. Element concentrations in the adipose tissue were generally lower and there were fewer significant effects, compared to the other two tissues (Tables 5 and 6). Caribou had higher concentrations of elements than muskox with significant differences, including Na, S, Mg, Zn, As and Cu (*p* < 0.05).

**Table 1:** Mean element concentration (mg/kg dry matter ± standard deviation) in the *longissimus dorsi* tissue of muskox and caribou. Absent means were found to be below quantification limits

Element class	Element	Muskox		Caribou	
		Male	Female	Male	Female
Macro	Ca	956.59 ± 368	1395.01 ± 1463	463.30 ± 69	470.91 ± 76
	K	5987.99 ± 1488	5880.01 ± 1584	8963.72 ± 1089	8619.16 ± 1100
	Mg	695.20 ± 94	655.28 ± 65	773.68 ± 75	832.28 ± 72
	Na	2396.85^a^ ± 480	2724.08^ab^ ± 572	3018.92^b^ ± 442	2654.39^ab^ ± 433
	P	4732.21 ± 827	4755.43 ± 730	6603.68 ± 700	6998.33 ± 653
	S	4216.93 ± 558	4226.21 ± 548	8220.79 ± 893	8276.74 ± 733
Micro	Co	1.08 ± 0.1	1.09 ± 0.1	1.14 ± 0.2	1.12 ± 0.1
	Cu	8.27 ± 1.8	9.97 ± 2.3	8.90 ± 1.2	11.01 ± 2.1
	Fe	94.79 ± 19	106.51 ± 20	147.64 ± 51	149.20 ± 49
	Mn	1.82 ± 0.9	1.78 ± 0.6	2.50 ± 0.5	3.12 ± 0.6
	Zn	78.98 ± 20	98.97 ± 48	148.76 ± 37	120.92 ± 50
Others	As	2.36 ± 0.3	2.48 ± 0.3	2.28 ± 0.3	2.30 ± 0.3
	Ba	3.83 ± 0.5	4.23 ± 1.0	5.17 ± 0.7	5.03 ± 1.0
	Cd				0.05 ± 0.00
	Cr		4.46 ± 1.7	2.67 ± 0.0	2.67 ± 0.9
	Li	2.50 ± 1.1	3.44 ± 1.0	1.97 ± 1.3	2.96 ± 1.4
	Pb	0.69 ± 0.1	0.80 ± 0.1	0.62 ± 0.1	4.04 ± 8.1
	Sn	3.16 ± 1.1	3.21 ± 0.5	2.94 ± 0.4	3.56 ± 0.5

Different letters indicate statistically significant differences with interaction

**Table 2:** Significant levels (*p*-values) obtained for the effects of species, sex and their interaction in the *longissimus dorsi* muscle of muskox and caribou

Class	Element	Species	Sex	Species × Sex	Observations per group			
					Muskox		Caribou	
					Male	Female	Male	Female
Macro	Ca	**0.0161**	0.4309	0.4467	8	12	10	8
	K	** < 0.0001**	0.6147	0.7921	8	12	10	8
	Mg	** < 0.0001**	0.7096	0.0558	8	12	10	8
	Na	0.0981	0.9092	**0.0405**	8	12	10	8
	P	** < 0.0001**	0.3897	0.4440	8	12	10	8
	S	** < 0.0001**	0.8875	0.9193	8	12	10	8
Micro	Co	0.4571	0.9725	0.7670	3	8	3	2
	Cu	0.1979	**0.0050**	0.7447	8	12	10	8
	Fe	**0.0004**	0.5915	0.6813	8	12	10	8
	Mn	** < 0.0001**	0.1762	0.1285	8	11	10	8
	Zn	**0.0019**	0.7750	0.0878	8	12	10	8
Others	As	0.2690	0.5288	0.6870	7	11	6	7
	Ba	**0.0005**	0.6452	0.3388	8	12	10	8
	Cd				0	0	0	1
	Cr	0.3220	0.9979		0	2	1	2
	Li	0.3249	0.0693	0.9577	7	7	4	5
	Pb	0.3485	0.2986	0.3274	7	10	3	6
	Sn	0.8120	0.2260	0.2939	7	11	5	6

Bold numbers represent statistical significance (*p *< 0.05)

**Table 3:** Mean element concentration (mg/kg dry matter ± standard deviation) in the liver tissue of muskox and caribou. Absent means were found to be below quantification limits

	Element	Muskox		Caribou	
		Male	Female	Male	Female
Macro	Ca	726.38 ± 91	716.28 ± 111	488.97 ± 110	470.36 ± 32
	K	4894.66 ± 594	5090.91 ± 482	6880.66 ± 475	6763.07 ± 406
	Mg	629.43 ± 55	606.05 ± 34.8	560.11 ± 51	548.26 ± 13
	Na	5456.53 ± 1046	5836.83 ± 1022	3116.87 ± 506	3162.29 ± 379
	P	8584.17 ± 483	8334.65 ± 850	9573.76 ± 572	9422.17 ± 645
	S	5921.59 ± 309	5822.80 ± 266	6806.40 ± 550	6730.84 ± 635
Micro	Co	1.46 ± 0.3	1.55 ± 0.3	1.17 ± 0.1	1.16 ± 1.1
	Cu	279.88 ± 90	251.61 ± 73	177.94 ± 91	135.63 ± 43
	Fe	621.74 ± 112	684.86 ± 150	431.50 ± 107	548.48 ± 230
	Mn	7.85 ± 1.2	8.39 ± 2.0	14.16 ± 2.9	14.62 ± 4.5
	Zn	65.34 ± 5.9	62.72 ± 5.7	62.58 ± 24	49.95 ± 7.1
Others	As	2.54 ± 0.2	2.53 ± 0.2	2.20 ± 0.1	2.17 ± 0.1
	Ba	4.14 ± 0.5	3.94 ± 0.5	5.74 ± 0.3	5.84 ± 0.3
	Cd	0.14 ± 0.04	0.14 ± 0.03	0.43 ± 0.2	0.50 ± 0.2
	Cr	9.26 ± 14	26.57 ± 27		
	Li	5.33 ± 1.5	5.24 ± 3.4	2.60 ± 0.8	3.02 ± 3.1
	Pb	0.96 ± 0.2	0.87 ± 0.2	0.75 ± 0.3	0.91 ± 0.2
	Sn	3.92 ± 0.5	3.37 ± 0.7	3.30 ± 0.6	3.07 ± 0.5

**Table 4:** Significant levels (*p*-values) obtained for the effects of species, sex and their interaction in the liver tissue of muskox and caribou

Class	Element	Effects			Observations per group			
		Species	Sex	Species × Sex	Muskox		Caribou	
					Male	Female	Male	Female
Macro	Ca	** < 0.0001**	0.6526	0.8937	8	12	9	8
	K	** < 0.0001**	0.8119	0.3454	8	12	9	8
	Mg	** < 0.0001**	0.212	0.6797	8	12	9	8
	Na	** < 0.0001**	0.4419	0.5445	8	12	9	8
	P	** < 0.0001**	0.3803	0.8294	8	12	9	8
	S	** < 0.0001**	0.5654	0.9388	8	12	9	8
Micro	Co	**0.0002**	0.6733	0.5682	8	12	8	8
	Cu	**0.0002**	0.1763	0.7853	8	12	9	8
	Fe	**0.0034**	0.0913	0.6064	8	12	9	8
	Mn	** < 0.0001**	0.5988	0.9636	8	12	9	8
	Zn	0.0885	0.0940	0.2655	8	12	9	8
Others	As	** < 0.0001**	0.7574	0.8529	8	12	6	3
	Ba	** < 0.0001**	0.6952	0.2947	8	12	9	8
	Cd	** < 0.0001**	0.4837	0.4407	8	12	9	8
	Cr		0.1521		7	9	-	-
	Li	**0.0465**	0.8876	0.8307	8	12	4	3
	Pb	0.2916	0.6378	0.1161	7	11	9	8
	Sn	**0.0462**	0.0879	0.4769	6	7	7	7

Bold numbers represent statistical significance (*p *< 0.05)

**Table 5:** Mean element concentration (mg/kg dry matter ± standard deviation) in the adipose tissue of muskox and caribou. Absent means were found to be below quantification limits

	Element	Muskox		Caribou	
		Male	Female	Male	Female
Macro	Ca	338.77 ± 82	405.13 ± 207	317.15 ± 57	374.10 ± 58
	K	272.05 ± 24	196.09 ± 0.0	382.17 ± 163.9	1583.87 ± 2311.67
	Mg	77.32 ± 6.9	78.27 ± 12	86.44 ± 18	158.19 ± 135
	Na	844.22^a^ ± 204	976.60^a^ ± 171	1041.25^a^ ± 240	1845.01^b^ ± 951
	P	96.31 ± 53	149.54 ± 111	213.31 ± 138	1390.37 ± 2513
	S	203.12 ± 44	215.95 ± 71	380.52 ± 150	1120.36 ± 1264
Micro	Co	1.14 ± 0.2	1.13 ± 0.1	1.18 ± 0.1	1.32 ± 0.2
	Cu	5.61 ± 1.7	7.03 ± 1.0	7.21 ± 1.7	8.20 ± 0.9
	Fe	23.85 ± 6.9	43.03 ± 43	29.13 ± 10	93.61 ± 106
	Mn	1.57 ± 0.6	1.71 ± 0.6	2.00 ± 0.6	2.04 ± 0.9
	Zn	2.56 ± 0.8	3.07 ± 0.6	4.39 ± 1.3	10.65 ± 14
Others	As	2.02 ± 0.01	2.00 ± 0.0	2.10 ± 0.1	2.32 ± 0.2
	Ba	4.40 ± 0.8	3.98 ± 0.4	4.3 ± 0.4	4.4 ± 0.5
	Cd				0.10 ± 0.0
	Cr	3.45 ± 0.0	7.96 ± 10		
	Li	2.48 ± 1.5	2.15 ± 0.3	3.48 ± 0.2	2.51 ± 1.2
	Pb	0.97 ± 0.1	0.70 ± 0.2	0.91 ± 0.5	121.56 ± 193
	Sn	3.93 ± 1.0	3.73 ± 0.9	4.26 ± 2.0	3.41 ± 0.8

Different letters indicate statistically significant differences with interaction.

**Table 6:** Significant levels (*p*-values) obtained for the effects of species, sex and their interaction in the adipose tissue of muskox and caribou

Class	Element	Effects			Observations per group			
		Species	Sex	Species × Sex	Muskox		Caribou	
					Male	Female	Male	Female
Macro	Ca	0.5889	0.2104	0.9228	8	12	8	6
	K	0.4470	0.5654	0.5151	2	1	7	5
	Mg	**0.0330**	0.0779	0.0857	8	12	8	6
	Na	**0.0015**	**0.0044**	**0.0349**	8	12	8	6
	P	0.0723	0.1018	0.1336	8	12	8	6
	S	**0.0065**	0.0508	0.0587	8	12	8	6
Micro	Co	0.0712	0.2963	0.2576	5	7	5	5
	Cu	**0.0068**	**0.0173**	0.6501	8	12	8	6
	Fe	0.1297	**0.0268**	0.2210	8	12	8	6
	Mn	0.1768	0.7320	0.8572	6	9	7	5
	Zn	**0.0301**	0.1120	0.1742	8	12	8	6
Others	As	**0.0320**	0.2174	0.1483	2	1	4	3
	Ba	0.3840	0.3828	0.1703	8	12	8	6
	Cd				-	-	-	1
	Cr		0.6955		1	6	-	-
	Li	0.3639	0.3807	0.6656	3	2	2	5
	Pb	0.0828	0.0838	0.0825	5	8	8	5
	Sn	0.9951	0.2500	0.4681	8	12	7	6

Bold numbers represent statistical significance (*p* < 0.05)

**Fig. 2:**
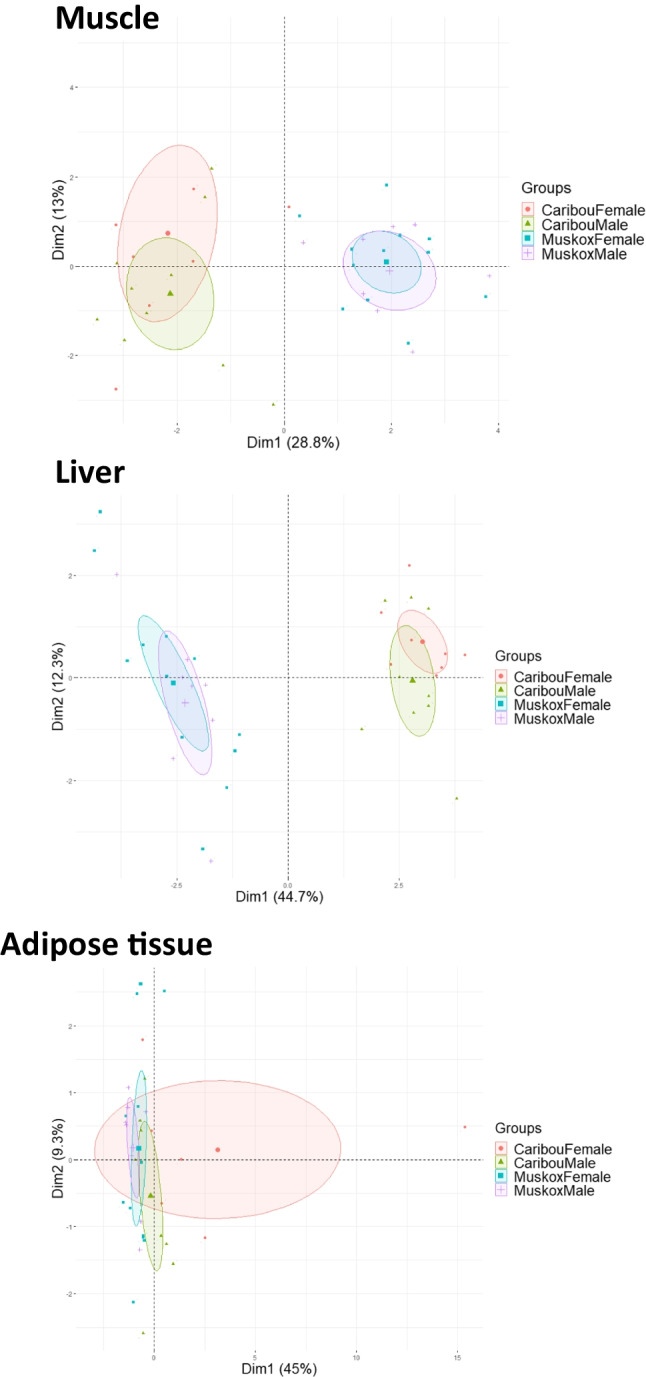
Principal component analysis for the element profile of the muscle, liver and adipose tissue of muskox and caribou

The differences mentioned above are corroborated by the PCA analysis performed (Fig. 2). Indeed, the effect of species is most pronounced in both the muscle and liver, with the adipose tissue having less discrimination between caribou and muskox. Distinction based on sex is not clearly recognizable.

The lower number of differences found in adipose tissue could point towards influence of muscle and hepatic lipid contents on those tissues’ profiles, given that fat has a diluting effect on tissue element profiles [7]. Excluding lipid content, the differences mentioned above could derive from multiple factors. Gamberg et al. [12] have analysed contaminants in caribou tissues from the KS and AM regions. According to these authors, in those regions, available forage is composed by monocots (KS) and lichens during winter (AM). Thus, in the KS area, muskoxen graze mostly on the available grasses, shrubs and other graminoids [13, 14]. In turn, the caribou of the present study can feed on lichens, which have been described to have a Ca concentration more than double the amount found in other feed sources such as *Salix* sp., upon which the muskox also rely [13, 15]. Lichen availability has been reported as being a major contributing factor for the element status of muscle and liver of the caribou [16]. It is expected that the muskox on the Kangerlussuaq area graze on graminoids to a larger extent than caribou in the AM region, which has a more diverse diet, including lichen depending on the time of year [17]. This could explain the higher content of hepatic Cu in the muskox, which corroborates the results found by Gamberg et al. [12] that state that caribou from KS have higher hepatic Cu accumulation as a result of dietary graminoids of higher Cu tolerance than lichens. Therefore, it could be suggested that the combined influence of diet and geographical location are major factors affecting these element profiles.

Caribou had significantly higher Cd concentration in the liver, compared to the muskox. In contrast, Cr was detected in the muskox liver and not in the caribou. A similar hepatic accumulation of heavy metals has been reported before in the Canadian Yukon [18], as the local caribou feed heavily on lichen that accumulates heavy metals. The same could apply in this study. However, the concentrations are low, since Greenland has comparatively lower rates of anthropogenic pollution [16]. Nonetheless, edible animal tissues should be monitored for their contaminating elements. Contaminants have been detected in other Arctic mammals such as the hare and ringed seal, derived from pollution coming from Eurasia [19–21]. For instance, Johansen et al. [19] has found that liver, kidney and blubber (of marine mammals) of arctic animals have the proclivity to be sources of contaminants, such as mercury (Hg) and Cd, despite ungulate tissues having predominantly very low to medium levels of these elements (non-hazardous levels), according to the authors’ classification.

In the muscle and adipose tissue, Cu was significantly influenced by sex, with females having higher concentrations than males. Our values on the female caribou muscle are within range from those reported in caribou females from AM (8 mg/kg DM) and KS (13 mg/kg DM) regions [12]. Interestingly, there were no significant effects of sex on Cu levels in the hepatic tissue, a main storage site of this element. Copper is an important co-factor for several enzymes such as cytochrome oxidase that participate in oxidation–reduction pathways [22]. It has been previously reported that female muskoxen upregulate these pathways [11]. Ljubojević et al. [23] have found that higher Fe in the liver of female rats is related to metallothionein synthesis and is accompanied by increased malondialdehyde (results from lipid peroxidation) levels, which could be related to increased oxidative stress. In the present study, female ungulates had higher Fe in both muscle and adipose tissue, although without statistical significance in the muscle. By having higher oxidative stress, female ungulates could upregulate oxidation–reduction pathways. The precise reason why these differences have occurred is unclear, and further research could benefit from using a more consistent set of samples (e.g. balanced groups) in order to further explain these discrepancies.

## Conclusions and Future Perspectives

The present study provides a glimpse into the element profile of edible tissues of West Greenland caribou and muskoxen, demonstrating differences between the two species. It is apparent that of the two factors “species” and “sex”, the former has the greatest impact on element composition of the three tested tissues, as confirmed in the PCA analysis. This likely reflects species-specific behaviour and physiology. Indeed, future research on these topics and food safety assessments should also include contaminant analyses (e.g. Pb, Cd and As) as different species accumulate different levels of contaminants. It is noteworthy for future studies to also consider, e.g. sampling animals harvested in the same region during the same season. Moreover, studying element concentrations relative to fat-free weight could eliminate diluting effects, since fat content has an influence on tissue element concentration.

## Supplementary Information

Below is the link to the electronic supplementary material.

Supplementary file1 (DOCX 14 KB)
