# Supplementary material for: The Effect of Species and Sex on the Element Content of Muskox (Ovibos moschatus) and Caribou (Rangifer tarandus groenlandicus) Tissues
Source: Biol Trace Elem Res. 2023 Jan 17;201(10):4718–25. doi: 10.1007/s12011-023-03562-x (PMC10415418; doi:10.1007/s12011-023-03562-x)
Supplement: Supplementary file 1 — Supplementary file1 (DOCX 14 KB) [file 12011_2023_3562_MOESM1_ESM.docx]

Supplementary material 1 - Recovery values of Certified Reference Materials (Wepal IPE 776) that underwent the same experimental procedure as the samples.

| **Element** | **% recovery** |
| --- | --- |
| Na | 95.7 |
| K | 94.1 |
| Ca | 97.5 |
| Mg | 101.2 |
| P | 102.3 |
| S | 95.2 |
| Cu | 101.8 |
| Mn | 98.7 |
| Fe | 94.7 |
| Zn | 98.5 |
| Co | 91.8 |
| As | 93.5 |
| Ba | 92.8 |
| Sn | 92.6 |
| Li | 91.7 |
| Cr | 93.5 |
| Cd | 98.3 |
| Pb | 96.2 |
